# Molecular Characterization of a Novel Polerovirus Infecting Soybean in China

**DOI:** 10.3390/v14071428

**Published:** 2022-06-29

**Authors:** Tengzhi Xu, Lei Lei, Yong Fu, Xiaolan Yang, Hao Luo, Xiangru Chen, Xiaomao Wu, Yaqin Wang, Meng-ao Jia

**Affiliations:** 1 Institute of Crop Protection, College of Agriculture, Guizhou University, Guiyang 550025, China; xtz9012@126.com (T.X.); fy0308@163.com (Y.F.); yxl1231@163.com (X.Y.); lh0805@163.com (H.L.); xrchen3@gzu.edu.cn (X.C.); wuxm827@126.com (X.W.); 2 Guizhou Rapeseed Institute, Guizhou Academy of Agricultural Sciences, Guiyang 550008, China; rainbow20053787@163.com; 3 State Key Laboratory of Rice Biology Institute of Biotechnology, Zhejiang University, Hangzhou 310058, China; 4 Guizhou Academy of Tobacco Sciences, Guiyang 550001, China

**Keywords:** soybean chlorotic leafroll virus, soybean, *Polerovirus*, high-throughput sequencing

## Abstract

Poleroviruses are positive-sense, single-stranded viruses. In this study, we describe the identification of a novel polerovirus isolated from soybean displaying curled leaves. The complete viral genome sequence was identified using high-throughput sequencing and confirmed using rapid amplification of cDNA ends (RACE), RT-PCR and Sanger sequencing. Its genome organization is typical of the members of genus *Polerovirus*, containing seven putative open reading frames (ORFs). The full genome is composed of single-stranded RNA of 5822 nucleotides in length, with the highest nucleotide sequence identity (79.07% with 63% coverage) for cowpea polerovirus 2 (CPPV2). Amino acid sequence identities of the protein products between the virus and its relatives are below the threshold determined by the International Committee of Taxonomy of Viruses (ICTV) for species demarcation, and this strongly supports this virus’ status as a novel species, for which the name soybean chlorotic leafroll virus (SbCLRV) is proposed. Recombination analysis identified a recombination event in the ORF5 of the 3’ portion in the genome. Phylogenetic analyses of the genome and encoded protein sequences revealed that the new virus is closely related to phasey bean mild yellows virus, CPPV2 and siratro latent polerovirus. Subsequently, we demonstrated the infectivity of SbCLRV in *Nicotiana benthamiana* via infectious cDNA clone generation and agroinoculation.

## 1. Introduction

*Solemoviridae* is a recently recognized plant-infecting virus family divided into four genera, named *Enamovirus, Polemovirus, Polerovirus* and *Sobemovirus*. The family members are characterized by a single-stranded positive-sense RNA genome of 4–4.6 kb in length and an icosahedral (t = 3) virion with a diameter of 26–34 nm [1]. *Polerovirus* contains the most species among the four genera [1]. The typical polerovirus genome is organized by seven ORFs, with a viral protein genome linked (VPg) covalently linked to the 5′-end. ORF0 encodes the viral silencing suppressor (VSR) [2]. ORF1 encodes P1, which contains the VPg and serine proteinase domain [3,4]. In addition, ORF1 could be expressed with ORF2 in a −1 ribosomal frameshift manner as a viral RNA-dependent RNA polymerase (RdRp) [5]. ORF3 and ORF4 are expressed by translation from subgenomic RNAs to encode coat protein (CP) [6,7] and movement protein (MP), respectively [8]. ORF5 is expressed via translational readthrough of the leaky stop codon of ORF3 to produce the putative P3–P5 fusion protein, which is necessary for vector transmission of the viral component [9]. A short non-AUG-initiated ORF, termed ORF3a, is located in the upstream of ORF3 and is involved in the viral long-distance movement and systemic infection of plants [10,11].

Soybean (*Glycine max* L.) is an economically important oilseed crop and vegetable-protein resource. The soybean crops are constantly threatened by diverse viruses, such as soybean mosaic virus (SMV), cucumber mosaic virus (CMV) and bean common mosaic virus (BCMV), resulting in global yield reduction. SMV is the most devastating and widely distributed virus in China [12]. In recent years, increasingly novel viruses were identified by deep sequencing virus-derived small-interfering RNAs (siRNA). followed by de novo assembly of the complete genome. The accurate identification of new viruses or novel virus strains is important for the control of viral pandemics. Here, soybean leaves showing symptoms of viral infection were collected from the Guizhou province of China. A virus detected by high-throughput sequencing was determined to be associated with this symptom. The sequence analysis and phylogenetic results strongly suggested that the virus is a novel species in the *Polerovirus* genus. Thus, we have provisionally named it soybean chlorotic leafroll virus (SbCLRV). To our knowledge, this is the first polerovirus reported to infect soybean in China. 

## 2. Materials and Methods

### 2.1. Sample Collection and RNA Extraction

The soybean leaves displaying virus-like symptoms were collected from the field in Guizhou province in China. For the viral genome sequencing and genome amplification, total RNA isolation was performed with TRIzol reagent (Tiangen, Beijing, China). The RNA integrity and quality were assessed via gel electrophoresis and absorbance OD260/280 nm using the nanodrop spectrometer, respectively. Good-quality RNA samples were thereby available for the subsequent research. 

### 2.2. Small RNA Library Construction, Sequencing and Data Processing

For small RNA-sequencing, TruSeq Small RNA Sample Preparation Kit (Illumina, San Diego, CA, USA) was used for the library construction. Briefly, 3′ and 5′ adapters were first ligated to the RNAs, followed by reverse transcription and amplification to construct the cDNA library. Based on the band size, the adapter-ligated small RNA fragments were purified from the PAGE gel as the concentrated final library. The qualified library was submitted to HiSeq 2000 platform (Illumina, Inc., CA, USA) for further analyses. The generated clean reads were mapped to the GenBank Virus RefSeq database using bowtie [13]. Virus reference genomes with the most abundant sRNA reads mapped were obtained. Meanwhile, contigs assembled through SPAdes [14] and velvet [15] processing methods were blasted against the reference genomes and annotated. 

### 2.3. Full-Length Genome Amplification, Sanger Sequencing

First-strand cDNA synthesis was performed with SuperScript II Reverse Transcriptase Kit (Invitrogen, Carlsbad, CA, USA) according to the supplier’s instructions. Briefly, incubation temperature was 42 °C for 30 min, followed by 15 min at 70 °C to stop the reaction and 20 min at 37 °C in presence of RNaseH (Invitrogen, Carlsbad, CA, USA) to degrade the RNA. The 5′- and 3′-terminal sequence of the genomic RNAs were determined by 5′- and 3′-rapid amplification of cDNA ends (RACE) experiments using the 5′/3′ RACE Kit, 2nd Generation (Roche, Germany) according to the manufacturer’s protocol. The specific primers for cDNA synthesis and RACE (primer pairs for the whole genome generation) were designed based on the assembled contigs (Table S1). PCR amplifications were performed with Pfu DNA Polymerase (Promega Inc, Madison, WI, USA). For Sanger sequencing, the fragments of expected size were purified with E.Z.N.A. Gel Extraction Kit (Omega Bio-tek, Norcross, GA, USA) or E.Z.N.A. Cycle Pure Kit (Omega Bio-tek, Norcross, GA, USA) for nucleic acids containing agarose gel purification or common PCR products purification, respectively. The purified fragments were cloned into pEASY-Blunt Cloning vector (TransGen, Beijing, China) and sequenced commercially (Qingke Biotech, Chongqing, China) for sequence validation with the common primer pairs: M13-47/M13-48 (Table S1). 

### 2.4. Viral Genome Characterization

The whole genome sequences were assembled with the aid of CLC Genomics Workbench 21.0.1 (Qiangen, Hilden, Germany) and DNAMAN software version 9.0 (Lynnon Biosoft, QC, Canada). Nucleotide sequences of ORFs on the genome were translated into amino acid sequences. The nucleotide and amino acid sequences of other poleroviruses were retrieved from the Genbank database in the National Center for Biotechnology Information (NCBI) (https://www.ncbi.nlm.nih.gov/, accessed on 19 May 2022). The newly assembled complete genome sequence was submitted to the Genbank database in NCBI through BankIt. 

### 2.5. Phylogenetic Analyses

For each polerovirus, one representative isolate was selected for analysis. ClustalW method was used for multiple sequence alignments. Phylogenetics tree construction was performed using MEGA 11.0.10 software (for windows) with maximum-likelihood (ML) algorithms [16], and the statistical confidence of branching was estimated via bootstrap analysis with 1000 replications. The SDT v1.2 software was used to display the pairwise identity of the ORFs aligned by the ClustalW program [17]. RDP4.3 software was used for the recombination events analyses among the distinct poleroviruses [18].

### 2.6. Construction of SbCLRV Infectious Clone and Agroinfiltration 

For construction of the infectious clone, four fragments covering the SbCLRV full-length genomic RNA were amplified and inserted into the pCass-RZ binary vector using the ClonExpress MultiS One Step Cloning Kit (Vazyme, Nanjing, China). Four primer pairs (Table S1) with homology arms were designed through Vazyme CE design website (https://crm.vazyme.com/cetool/simple.html, accessed on 21 December 2021). PCR amplifications were performed with Pfu DNA Polymerase (Promega Inc, Madison, USA). The 25 uL PCR mixture contains 2 uL cDNA, 2.5 uL Pfu DNA Polymerase 10* buffer, 2 uL dNTP (2.5 mM), 1ul forward/reverse primer (10 uM), 0.5 uL Pfu DNA Polymerase and 16 ul distilled water. The amplification was carried out under the following isothermal conditions: (1) 3 min denaturation at 95 °C; (2) 25 cycles, each of 30 s at 95 °C, 30 s at 55 °C and 1–3 min at 72 °C; (3) 10 min final extension at 72 °C. The resulting PCR fragments were mixed in a certain quality with linearized pCass-RZ vector (gifted from Prof. Chenggui Han, China Agricultural University) using Exnase MultiS, incubated at 37 °C for 30 min and transformed into the DH5α competent cells. Using colony PCR and Sanger sequencing, we screened the positive clone of pCass-SbCLRV.

For agroinfiltration, we first introduced the pCass-SbCLRV into the Agrobacterium tumefaciens strain GV3101. The transformed agrobacteria were infiltrated into the abaxial surface of the Nicotiana benthamiana seedlings leaves. Mock infiltrations were conducted with the pCass-RZ empty vector-transformed A. tumefaciens cells. The inoculated plants were kept in a greenhouse under condition 16/8 h (light/dark) photoperiod for symptoms to develop. After obvious symptoms developed in the systemically infected leaves, symptomatic tissues were detected by RT-PCR with viral-specific primers pXT297/pXT298 (Table S1). *NbActin* was used as an internal control to normalize the RNA level [19]. PCR products were analyzed on a 2% agarose gel.

### 2.7. Strand-Specific RT-PCR

RT-PCR was used for the strand-specific detection of positive- and negative-strand SbCLRV RNA. For each RNA, two RT reactions were performed with SuperScript II Reverse Transcriptase Kit (Invitrogen, Carlsbad, CA, USA) with specific primer pXT297 or pXT298, following the manufacturer’s instructions. Two microliters of cDNA were used as template for the PCR reaction in presence of primers pXT297 and pXT298, which produce a 1150 bp fragment. A seminested PCR was carried out using 1 ul of the first PCR run product as template, with the primer pXT297 and pXT299, to generate a 238 bp fragment. 

## 3. Results

### 3.1. Identification of Novel Virus through High-Throughput Sequencing

**Figure 1:**
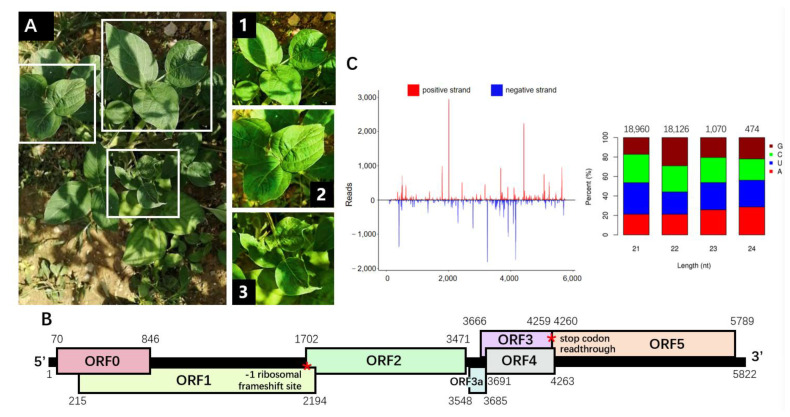
Characterization of the soybean chlorotic leafroll virus (SbCLRV) genome. (**A**) The chlorotic and leafroll symptoms represented on the soybean plant infected by SbCLRV. An overall view of the whole plant. Closer photographs (1, 2 and 3) focus on the symptomatic leaves, which showed curling in all the leaves (1, 2 and 3) and chlorosis in the downward leaves (3). (**B**) Schematic representation of the genomic organization of the SbCLRV. The positions of −1 ribosomal frameshift site in ORF1 and the stop codon for ORF3 readthrough were marked as red asterisks. (**C**) Distribution profile of the vsiRNAs that revealed multiple hotspots along the SbCLRV genome. The red and blue color represent the reads on the viral strand or viral complementary strand, respectively. The 5′-nucleotide bias of the vsiRNAs that matched the SbCLRV genomic sequence has been detailed in the histogram. Histogram bars represent the percentage of each of the four nucleotides, and the color code is detailed in the legend.

During a field survey conducted in July 2021 in a pepper and soybean intercropping field located in Guizhou (China), a soybean seedling with virus-like symptom was observed (Figure 1A). The infected leaves showed curling, and the downward leaves exhibited mild chlorosis. 

The HTS method was used to verify the viral agent. We constructed the small RNA library, which was then submitted to the Illumina Hiseq^TM^ 2000 platform for analysis. A total of 14,174,269 clean reads were generated. We removed the host sequences by mapping against the *Glycine max* reference genome and screening out the reads of 18 to 26 nt in length for the subsequent viral siRNA analyses. Finally, 32,468 reads were mapped to the Genbank Virus RefSeq. De novo assemblies were performed using SPAdes and velvet methods, which produced 440 and 394 contigs and ranged from 172 to 3230 nt in length, respectively. Most of the contigs were annotated as cowpea polerovirus 2 (CPPV2) and phasey bean mild yellows virus (PBMYV). The contigs mapped to the polerovirus genomes showed lower nucleotide identities ranging from 45% to 87%, which indicated that the agent might be a novel polerovirus. 

### 3.2. Complete Sequence and Organization of SbCLRV Genome

To further confirm the HTS results and identify the complete virus genomic sequence, total RNA was extracted from the diseased leaves for the subsequent RACE and PCR experiments. Primers were designed, based on the assembled contig sequences, to generate viral genome amplicons with overlapping regions reserved (Table S1). By piecing and trimming, the complete genomic sequence of 5822 nt in length was obtained. A BLASTn search with the nucleotide sequence showed 63% coverage and 79.07% sequence identity for the CPPV2 isolate BE179 (KY364847.1) as the highest scoring hit, followed by 47% coverage and 82% sequence identity for several PBMYV isolates. Thus, we referred to these sequences for the genomic organization characterization. An ORF finder was applied for the open reading frames prediction and computational identification. Six large ORFs were found on the plus strand in three reading frames (Figure 1B), which showed a genomic organization typical of polerovirus. The predicted protein products were submitted to the BLASTp search, which returned results on known poleroviruses (Table 1), and further characterized. We provisionally named this new genome soybean chlorotic leafroll virus (SbCLRV) and deposited it in Genbank with the accession number OM507197.

ORF0 is positioned at 70–846 nt in the genome (Figure 1B), predicted to encode the 258 aa RNA silencing suppressor protein (P0) with a predicted molecular mass of 29.4 kDa. The amino acid sequence shares 44.07% identity (90% query coverage, 6 × 10^−54^ E-value) to the P0 of PBMYV (QTJ01847.1) (Table 1). P0 protein of polerovirus was reported to carry an F-box motif (LPxxI/L) to form the SCF-like complex, which is involved in the RNA silencing suppressor function [20]. As expected, we found a F-box-like motif in the P0 of SbCLRV without conservation of the P as LSLLL, which was located at 60 aa (Figure S1). ORF1 is located at 215–2194 nt position (Figure 1B), expected to encode the 659 aa P1 protein with a calculated molecular weight of 73.1 kDa. P1 is most similar (61.88% amino acid identity, 97% coverage and 0 E-value) to the P1 of CpCV2 (YP_009352253.1) (Table 1). Meanwhile, ORF1 is expected to yield a −1 ribosomal frameshift polypeptide by combination with ORF2 (Figure 1B). The −1 ribosomal frameshifting event is mediated by a putative slippery sequence (X XXY YYZ) [21]. We performed the slippery sequence prediction and found a G GGA AAC sequence located at the 1699 nt position, which connects ORF1 and 2 (215–1699, 1702–3471 nt) to encode a 1085 aa P1–P2 protein of 122.4 kDa. The P1–P2 protein is with the highest identity (73.63% amino acid identity, 97% coverage and 0 E-value) to the P1–P2 from CpCV2. ORF3 starts at 3666 nt and ends at 4259 nt (Figure 1B), encoding the coat protein P3 of 197 aa (21.9 kDa) with 88% identity (82.39% query coverage, 2 × 10^−84^ E-value) to P3 of PBMYV (QTJ01893.1) (Table 1). ORF5 positions in 4260–5789 nt and translates to an in-frame readthrough of P3 stop codon (4257–4259 nt) (Figure 1B). The resulting P3–P5 fusion protein (706 aa, 79.6 kDa) shares 59.38% identity (90% query coverage, 0 E-value) with siratro latent polerovirus (SLPV) P3-P5 (QBR53291.1) (Table 1). We found a C-rich sequence presented at the 5′ end of the ORF5, which encodes a typical proline-rich sequence downstream of the P3 stop codon. ORF4 (3691–4263 nt) encodes movement protein (P4) (Figure 1B), with high identity but low query coverage (98% amino acid identity, 56.15% coverage and 3 × 10^−63^ E-value) to P4 from CPPV2 (QXU64018.1) (Table 1). A non-canonical ORF, ORF3a, starts with start codon ATA (3548–3550 nt) and ends at 3685 nt (Figure 1B). The predicted SbCLRV P3a (45 aa, 4.9 kDa) is most similar (78% amino acid identity, 84% coverage and 2 × 10^−22^ E-value) to PBMYV P3a protein (QHI06645.1) (Table 1). In addition, the 5′-UTR (1–69 nt) and 3′-UTR (5790–5822 nt) were of 69 nt and 32 nt in length, respectively. 

### 3.3. Analysis of the Virus-Derived sRNAs

The vsiRNAs were mapped to the SbCLRV genome, which can be visualized in viral small RNA hotspots along the plot (Figure 1C). In the single nucleotide resolution map, vsiRNAs were distributed throughout the genome in positive and negative orientations. Size-class distribution of the mapping sRNA revealed a prevalence of 21 nt, followed by 22 nt (Figure 1C). Analysis of the 5′-nucleotides bias of the SbCLRV-derived sRNAs showed a prevalence for 21 nt with U, 22 nt with G (Figure 1C). 

### 3.4. Phylogenetic Relationship of SbCLRV with Other Poleroviruses

**Figure 2:**
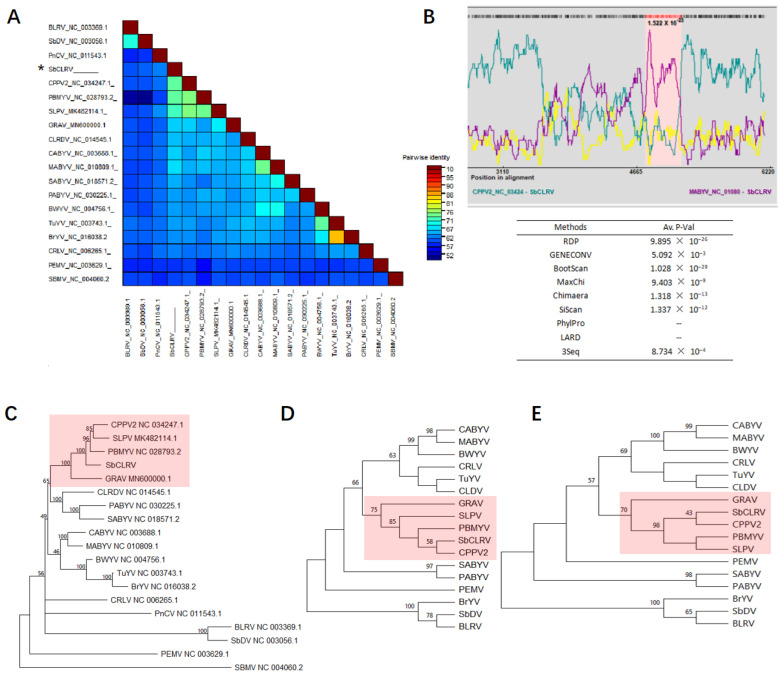
Phylogenetic analyses of the soybean chlorotic leafroll virus (SbCLRV) and selected members of the family *Solemoviridae.* (**A**) The pairwise identities plot of complete genomic nucleotide sequences aligned by MAFFT and displayed by sequence demarcation tool (SDT) software; (**B**) RDP3 analysis on the recombination event of these members. The peaks in pink zone represent the recombination events of SbCLRV with melon aphid-borne yellows virus and cotton leafroll dwarf virus in positions 5337–5578 nt and 5034–5255 nt, respectively; phylogenetic analyses of selected viruses based on the nucleotide sequences of (**C**) complete genome (model GTR + G + I) and amino acid sequences of the proteins (**D**) P1–P2 (RdRp) (model WAG + G) and (**E**) P3 (CP) (model JTT + G). The phylogenetic tree was constructed using the maximum likelihood (ML) method with 1000 bootstrap replications. The Genbank accession numbers for these genome sequences are listed in Table S3.

Several known polerovirus genome sequences were retrieved from the Genbank database for pairwise sequence identity analysis against SbCLRV using SDT software. The results showed that the pairwise sequence identities were relatively high between SbCLRV and PBMYV, CpCV2, SLPV or groundnut rosette assistor virus (GRAV), which ranged from 61.1% to 70.8% (Figure 2A, Table S2).

A recombination analysis performed with RDP4 on a multiple alignment of all these polerovirus genomic sequences revealed one recombination event between a CPPV2 major parent and a melon aphid-borne yellow virus (MABYV) minor one (Figure 2B). This recombination event was located in position 4781–5177 nt within the SbCLRV P5 cistron (ORF5) (Figure 2B). The result was supported from seven of nine programs used in this study, including RDP (p-value of 9.895 × 10^−26^), GENECONV (*p*-value of 5.092 × 10^−3^), Bootscan (*p*-value of 1.028 × 10^−29^), MaxChi (p-value of 9.403 × 10^−9^), Chimaera (*p*-value of 1.318 × 10^−13^), SiScan (*p*-value of 1.337 × 10^−12^) and 3Seq (*p*-value of 8.734 × 10^−4^) with the *p*-value < 10^−3^ (Figure 2B).

To better evaluate the relationship of SbCLRV within the *Polerovirus*, we constructed ML phylogenetic trees for analyses based on the amino acid sequences of P1–P2 (model WAG + G) (Figure 2D), P3 (model JTT + G) (Figure 2E) and the full-length genomic nucleotide sequences (model GTR + G + I) (Figure 2C). The SbCLRV was showed to be a distinct member of *Polerovirus*, more closely related to a subgroup composed of PBMYV, CPPV2, SLPV and GRAV in each of the phylogenetic tree (Figure 2C–E).

### 3.5. SbCLRV Infectivity in N. benthamiana Plants

**Figure 3:**
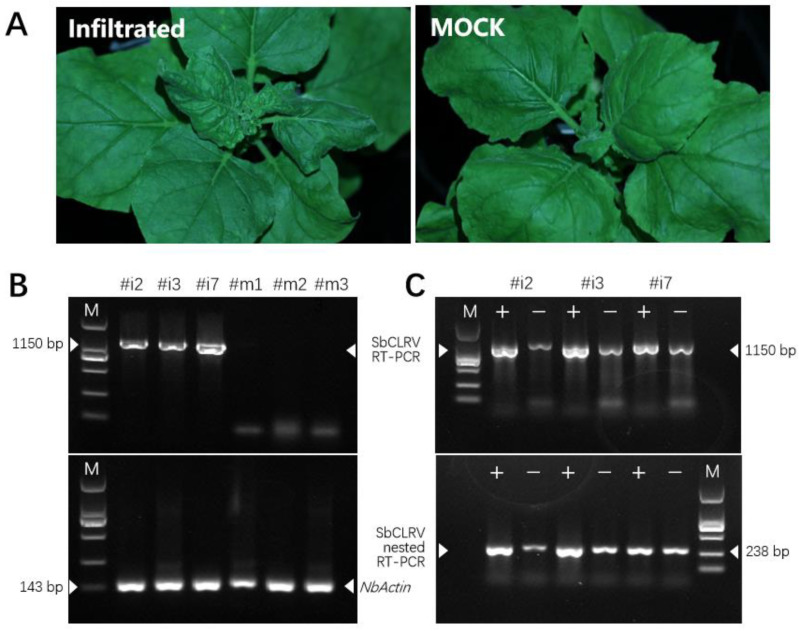
Symptoms on leaves of *Nicotiana benthamiana* infected by soybean chlorotic leafroll virus (SbCLRV) and virus detection. (**A**) Chlorosis and leafroll symptoms on upper leaves of *N. Benthamiana* inoculated by SbCLRV. Pictures were taken at 10 dpi; (**B**) RT-PCR detection of the symptomatic leaves (line 1, 2, 3) and mock-inoculated leaves (line 4, 5, 6) at 15 dpi; (**C**) detection of replicating SbCLRV by strand-specific RT-PCR and seminested RT-PCR. “+” and “−” represent the positive- and negative-strand-specific RT-PCR, respectively.

To investigate the infectivity of this novel virus, the full-length clone of SbCLRV was constructed as pCass-SbCLRV for agroinoculation assay. We infiltrated the recombinant *A. tumefaciens* cells into the *N. benthamiana* seedlings leaves. Ten days later, chlorosis and leaf curling symptoms showed on the upper leaves of the inoculated seedlings (Figure 3A). RT-PCRs with SbCLRV-specific primers were performed against the symptomatic upper leaves and shown to be positive (Figure 3B, Table S1). In addition, we performed the negative strand-specific RT-PCR and seminested RT-PCR to detect the replication of SbCLRV, since SbCLRV is a positive-strand virus and negative-strand RNA is only found when it is replicating. The replicating virus was detected in all of the positive samples (Figure 3C). These results indicate that SbCLRV could infect *N. benthamiana*.

## 4. Discussion

In this paper, a full-length sequence of soybean chlorotic leafroll virus (SbCLRV) was determined, which has all the genomic characteristics of polerovirus and groups with other poleroviruses in the phylogenetic trees. Polerovirus genome gathers several organization and expression strategies that especially identify SbCLRV. (i) The F-box motif (LPxxI/L) in P0 is required for silencing suppressor activity [20]. This motif of SbCLRV is present as LSLLL, which is not fully conserved. As shown in Figure S1, variations are always observed in this motif, such as LCFLLR in PBMYV and GRAV. However, the RNA silencing suppressor functions of these P0 proteins need to be further confirmed. (ii) The −1 ribosomal frameshift signal in ORF1/ORF2. The site is always associated with a slippery sequence (X XXY YYZ) and a downstream RNA pseudoknot [5,21,22]. (iii) Non-AUG-initiated ORF3a [10,11]. The amino acid sequence of P3a is conserved in residues as well as length (45–46 aa). (iv) A proline-rich sequence downstream of the P3 stop codon [23]. According to the International Committee of Taxonomy of Viruses (ICTV) [1], the species-demarcation criteria in the family *Solemoviridae* suggest that the amino acid sequence identity difference in any of the predicted protein products between two different species should be greater than 10%. The identities of all the SbCLRV-encoded proteins ranged from 44.07% to 98% to the best matched sequences, meeting with the criteria for new species. With this guideline, we propose that SbCLRV (Genbank accession no. OM507197) should be considered a new member within the genus *Polerovirus*. 

We analyzed the SbCLRV-derived vsiRNAs and found a majority of 21 and 22 nt vsiRNAs, which is consistent with previous reports on various virus-derived vsiRNA patterns [24,25,26], indicating that DCL4 and DCL2 play dominant roles in generating vsiRNAs. Different 5′-nucleotides of sRNAs are associated with special AGO recruitment preferences. AGO1 mainly recruits siRNAs with U at the 5′ end [27,28]. We found that the 21 nt vsiRNAs exhibited 5′-U bias, which indicated that they were prevalently recruited by the AGO1 complex. For 22 nt vsiRNA, 5′-G bias was observed, which has seldom been reported previously with a particular AGOs preference.

A potential recombination event for SbCLRV, with MABYV and CPPV2, was identified within the P5 cistron by recombination detection analysis. It has been reported that polerovirus P5 protein was highly diversified in the C-terminus but conserved in the N-terminus [29]. We confirmed this with a ClustalW alignment based on the P5 amino acid sequences among several poleroviruses (Figure S2). We found a dividing line between the conserved and variable sequences of P5 at the 227 aa (4940 nt located in the SbCLRV genome), which was located within the recombination region (4781–5177 nt), indicating that the recombination integrated both the N-conserved and C-variable ends (Figure S2). The recombination event associated with ORF5 seems to be common in many poleroviruses [23,30,31]. It could be assumed that the recombination with the P5 conserved sequences broadens the potential host-range since P5 is related to the aphid transmission, while recombination with the diversified sequences contributes to new polerovirus development [9]. Meanwhile, co-infections might result from the recombination. In this study, we found SbCLRV recombination associated with the CPPV2 major parent and a MABYV minor one. As inferred, the host range might be extended, although MABYV has not been reported to be hosted by legume plants [30]. Since RdRp and CP are conserved proteins in poleroviruses [24,31], we used their amino acid sequences for the phylogenetic analyses. Additionally, phylogenetic analysis based on complete genome sequences is necessary [23]. Using pairwise nucleotide sequence analysis and phylogenetic analyses, we found that SbCLRV is closely related to the PBMYV, SLPV and CPPV2, suggesting a close relationship between these legume crop-infecting poleroviruses. Apart from the significance of the disease induced by these viruses on the cultivation of legume, these will be an excellent case study of poleroviruses speciation and modular evolution [23].

It was known that polerovirus is obligatorily phloem-limited and relies on aphids for transmission [31]. These characters make it difficult to conduct further functional study on *Polerovirus* members. However, this difficulty has been conquered through the developments of many polerovirus cDNA infectious clones [23,24,32,33]. Usually, an infectious clone is constructed under the control of the cauliflower mosaic virus (CaMV) 35S promoter and fused with a ribozyme (RZ) for polyprotein cleavage. Moreover, agroinfection via *A. tumefaciens* harboring the infectious clone functions as an alternative procedure for aphids with regards to polerovirus infection. In our study, agroinoculation of the combinant pCass-SbCLRV on *N. benthamiana* produced the SbCLRV-like symptom, which demonstrated the SbCLRV infectivity. However, this agroinfection assay could not be reproduced in soybean, although legumes have been agroinfected. We will investigate whether this is the genotype of the soybean associated with the SbCLRV infectivity. In addition, further work should focus on diagnostic tool development for controlling and quarantining this novel virus. The host range, transmission methods and the underlined molecular mechanism are also worth investigation.

## 5. Conclusions

We found a soybean seedling with virus-like symptoms during a field survey in Guizhou, China. The causal agent was identified as a novel polerovirus based on genome and phylogenetic analyses, which was provisionally named soybean chlorotic leafroll virus (SbCLRV).

## Figures and Tables

**Table 1:** Top match from amino acid sequence comparisons with predicted proteins from soybean chlorotic leafroll virus through blastp searches of the NCBI non-redundant protein sequence database.

ORF	Protein	Predicted Function	Length (aa)	Top Match Protein Name, Organism, Accession Number (NCBI)	Amino Acid Identity (%)	Query Coverage (%)	E-Value
ORF0	P0	RNA silencing suppressor	258	phasey bean mild yellows virus QTJ01847.1	44.07	90	6.00 × 10^−54^
ORF1	P1	Multi-functional protein	659	cowpea polerovirus 2 YP_009352253.1	61.88	97	0
ORF1–2	P1–P2	RNA-dependent RNA polymerase	1085	cowpea polerovirus 2 YP_009352252.1	73.63	97	0
ORF3	P3	Coat protein	198	phasey bean mild yellows virus QTJ01893.1	88	82.39	2.00 × 10^−84^
ORF4	P4	Movement protein	191	cowpea polerovirus 2 QXU64018.1	98	56.15	3.00 × 10^−63^
ORF3–5	P3–P5	putative aphid Transmission factor	708	siratro latent polerovirus QBR53291.1	59.36	90	0
ORF3a	P3a	Long-distance transmission	45	phasey bean mild yellows virus QHI06645.1	78	84	2.00 × 10^−22^

## Supplementary Materials

The following supporting information can be downloaded at: https://www.mdpi.com/article/10.3390/v14071428/s1, Table S1. Primers used in RACE- or RT-PCR for amplification of whole genome or virus detection; Table S2. The SDT pairwise identities numbers of the nucleotide genomic sequence among the poleroviruses aligned by MAFFT; Table S3. The reference viruses used in phylogenetic analyses; Figure S1. Multiple alignment of the polerovirus P0 amino acid sequences encompassing a conserved F-box motif LPxxL/I. Figure S2. A dividing line between the N conserved- and C variable-sequences shown in the overview of multiplex sequence alignment of P5 among different poleroviruses.

## References

[1] Sõmera M., Fargette D., Hébrard E., Sarmiento C. (2021). Ictv Report Consortium. ICTV Virus Taxonomy Profile: Solemoviridae. J. Gen. Virol..

[2] Fusaro A.F., Correa R.L., Nakasugi K., Jackson C., Kawchuk L., Vaslin M.F., Waterhouse P.M. (2012). The Enamovirus P0 protein is a silencing suppressor which inhibits local and systemic RNA silencing through AGO1 degradation. Virology.

[3] van der Wilk F., Verbeek M., Dullemans A.M., van den Heuvel J.F. (1997). The genome-linked protein of potato leafroll virus is located downstream of the putative protease domain of the ORF1 product. Virology.

[4] Li X., Halpin C., Ryan M.D. (2007). A novel cleavage site within the potato leafroll virus P1 polyprotein. J. Gen. Virol..

[5] Nixon P.L., Cornish P.V., Suram V., Giedroc D.P. (2002). Thermodynamic analysis of conserved loop-stem interactions in P1-P2 frameshifting RNA pseudoknots from plant *Luteoviridae*. Biochemistry.

[6] Brault V., Bergdoll M., Mutterer J., Prasad V., Pfeffer S., Erdinger M., Richards K.E., Ziegler-Graff V. (2003). Effects of point mutations in the major capsid protein of beet western yellows virus on capsid formation, virus accumulation, and aphid transmission. J. Virol..

[7] Lee L., Kaplan I.B., Ripoll D.R., Liang D., Palukaitis P., Gray S.M. (2005). A surface loop of the potato leafroll virus coat protein is involved in virion assembly, systemic movement, and aphid transmission. J. Virol..

[8] Schmitz J., Stussi-Garaud C., Tacke E., Prufer D., Rohde W., Rohfritsch O. (1997). *In situ* localization of the putative movement protein (pr17) from potato leafroll luteovirus (PLRV) in infected and transgenic potato plants. Virology.

[9] Xu Y., Ju H.J., DeBlasio S., Carino E.J., Johnson R., MacCoss M.J., Heck M., Miller W.A., Gray S.M. (2018). A stem-loop structure in potato leafroll virus open reading frame 5 (ORF5) is essential for readthrough translation of the coat protein ORF stop codon 700 bases upstream. J. Virol..

[10] Smirnova E., Firth A.E., Miller W.A., Scheidecker D., Brault V., Reinbold C., Rakotondrafara A.M., Chung B.Y.W., Ziegler-Graff V. (2015). Discovery of a small non-AUG-initiated ORF in poleroviruses and luteoviruses that is required for long-distance movement. PLoS Pathog..

[11] Zhang X.Y., Zhao T.Y., Li Y.Y., Xiang H.Y., Dong S.W., Zhang Z.Y., Wang Y., Li D.W., Yu J.L., Han C.G. (2018). The conserved proline18 in the *Polerovirus* P3a is important for brassica yellows virus systemic infection. Front. Microbiol..

[12] Gao L., Sun S., Li K., Wang L., Hou W., Wu C., Zhi H., Han T. (2018). Spatio-temporal characterisation of changes in the resistance of widely grown soybean cultivars to Soybean mosaic virus across a century of breeding in China. Crop Pasture Sci..

[13] Langmead B., Trapnell C., Pop M., Salzberg S.L. (2009). Ultrafast and memory-efficient alignment of short DNA sequences to the human genome. Genome Biol..

[14] Sirotkin A., Vyahhi N., Tesler G., Alekseyev M.A., Pevzner P.A. (2012). SPAdes: A New Genome Assembly Algorithm and Its Applications to Single-Cell Sequencing. J. Comput. Biol..

[15] Zerbino D.R., Birney E. (2008). Velvet: Algorithms for de novo short read assembly using de Bruijn graphs. Genome Res..

[16] Tamura K., Stecher G., Kumar S. (2021). MEGA11: Molecular Evolutionary Genetics Analysis Version 11. Mol. Biol. Evol..

[17] Muhire B.M., Varsani A., Martin D.P. (2014). SDT: A virus classification tool based on pairwise sequence alignment and identity calculation. PLoS ONE.

[18] Martin D.P., Murrell B., Golden M., Khoosal A., Muhire B. (2015). RDP4: Detection and analysis of recombination patterns in virus genomes. Virus Evol..

[19] Li Y., Wang K., Lu Q., Du J., Wang Z., Wang D., Sun B., Li H. (2017). Transgenic Nicotiana benthamiana plants expressing a hairpin RNAi construct of a nematode *Rs-cps* gene exhibit enhanced resistance to *Radopholus similis*. Sci. Rep..

[20] Pazhouhandeh M., Dieterle M., Marrocco K., Lechner E., Ziegler-Graff V. (2006). F-box-like domain in the polerovirus protein P0 is required for silencing suppressor function. Proc. Natl. Acad. Sci. USA.

[21] Bankevich A., Nurk S., Antipov D., Gurevich A., Dvorkin M., Kulikov A.S., Lesin V., Nikolenko S., Pham S., Prjibelski A. (2014). Local structural and environmental factors define the efficiency of an RNA pseudoknot involved in programmed ribosomal frameshift process. J. Phys. Chem. B.

[22] Bock L.V., Caliskan N., Korniy N., Peske F., Rodnina M.V., Grubmüller H. (2019). Thermodynamic control of −1 programmed ribosomal frameshifting. Nat. Commun..

[23] Liu L., Ren Q., Peng B., Kang B., Wu H., Gu Q. (2022). Construction of an Agrobacterium-mediated infectious cDNA clone of melon aphid-borne yellows virus. Virus Res..

[24] Chen S., Jiang G., Wu J., Liu Y., Qian Y., Zhou X. (2016). Characterization of a novel polerovirus infecting maize in China. Viruses.

[25] Xia Z., Zhao Z., Chen L., Li M., Zhou T., Deng C., Zhou Q., Fan Z. (2016). Synergistic infection of two viruses MCMV and SCMV increases the accumulations of both MCMV and MCMV-derived siRNAs in maize. Sci. Rep..

[26] Li M., Li Y., Xia Z., Di D., Zhang A., Miao H., Zhou T., Fan Z. (2017). Characterization of small interfering RNAs derived from Rice black streaked dwarf virus in infected maize plants by deep sequencing. Virus Res..

[27] Mi S., Cai T., Hu Y., Chen Y., Hodges E., Ni F., Wu L., Li S., Zhou H., Long C. (2008). Sorting of small RNAs into *Arabidopsis argonaute* complexes is directed by the 5’ terminal nucleotide. Cell.

[28] Shen C., Wei C., Li J., Zhang X., Zhong Q., Li Y., Bai B., Wu Y. (2020). Barley yellow dwarf virus-GAV-derived vsiRNAs are involved in the production of wheat leaf yellowing symptoms by targeting chlorophyll synthase. Virol. J..

[29] Peter K.A., Gildow F., Palukaitis P., Gray S.M. (2009). The C terminus of the polerovirus p5 readthrough domain limits virus infection to the phloem. J. Virol..

[30] Lotos L., Olmos A., Orfanidou C., Efthimiou K., Avgelis A., Katis N.I., Maliogka V.I. (2017). Insights into the Etiology of Polerovirus-Induced Pepper Yellows Disease. Phytopathology.

[31] Zhao K., Yin Y., Hua M., Wang S., Mo X., Yuan E., Zheng H., Lin L., Chen H., Lu Y. (2021). Pod pepper vein yellows virus, a new recombinant polerovirus infecting Capsicum frutescens in Yunnan province, China. Virol. J..

[32] Wetzel V., Brault V., Varrelmann M. (2018). Production of a Beet chlorosis virus full-length cDNA clone by means of Gibson assembly and analysis of biological properties. J. Gen. Virol..

[33] Zhang P., Liu Y., Liu W., Cao M., Massart S., Wang X. (2017). Identification, Characterization and Full-Length Sequence Analysis of a Novel Polerovirus Associated with Wheat Leaf Yellowing Disease. Front. Microbiol..

