# Supplementary material for: Molecular Characterization of a Novel Polerovirus Infecting Soybean in China
Source: Viruses. 2022 Jun 29;14(7):1428. doi: 10.3390/v14071428 (PMC9322011; doi:10.3390/v14071428)
Supplement: Supplementary file 1 [file viruses-14-01428-s001.zip › viruses-1783399-figure S1.pdf]

Figure S1

	Species/Abbrv	□	□	□	□	□	□	□	□	□	□	□
*	1. Soybean chlorotic leafroll virus	V	F	L	L	S	L	L	L	R	G	P
	2. Suakwa aphid-borne yellows virus	I	F	L	L	P	L	C	L	N	P	L
	3. Melon aphid-borne yellows virus	L	A	R	L	P	L	L	I	S	E	Q
	4. Turnip yellows virus	L	Y	Q	L	P	L	L	L	G	G	H
	5. Cotton leafroll dwarf virus	L	F	L	L	P	F	F	I	R	G	I
	6. Brassica yellows virus	L	Y	Q	L	P	L	H	L	G	D	H
	7. Cucurbit aphid-borne yellows virus	L	A	R	L	P	L	L	I	S	E	Q
	8. Pepper vein yellows virus	C	A	L	L	P	F	L	L	S	S	K
	9. Siratro latent polerovirus	L	F	F	L	P	L	V	S	N	L	Q
	10. Groundnut rosette assistor virus	V	L	L	L	C	F	L	L	R	G	P
	11. Phasey bean mild yellows virus	L	F	L	L	C	F	L	L	R	G	P
	12. Beet western yellows virus	L	A	R	L	P	L	L	L	S	E	Q
	13. Carrot red leaf virus	L	Y	I	L	P	C	I	L	N	N	D
	14. Pepo aphid-borne yellows virus	L	F	L	I	P	I	A	L	R	S	D
	15. Cowpea polerovirus 2	V	F	L	L	P	F	L	L	R	G	P
	16. Pea enation mosaic virus-1	I	F	C	G	P	T	E	A	L	Q	R
