# Supplementary figures and images for: Molecular Characterization of a Novel Polerovirus Infecting Soybean in China
Source: Viruses. 2022 Jun 29;14(7):1428. doi: 10.3390/v14071428 (PMC9322011; doi:10.3390/v14071428)

## Figure S2

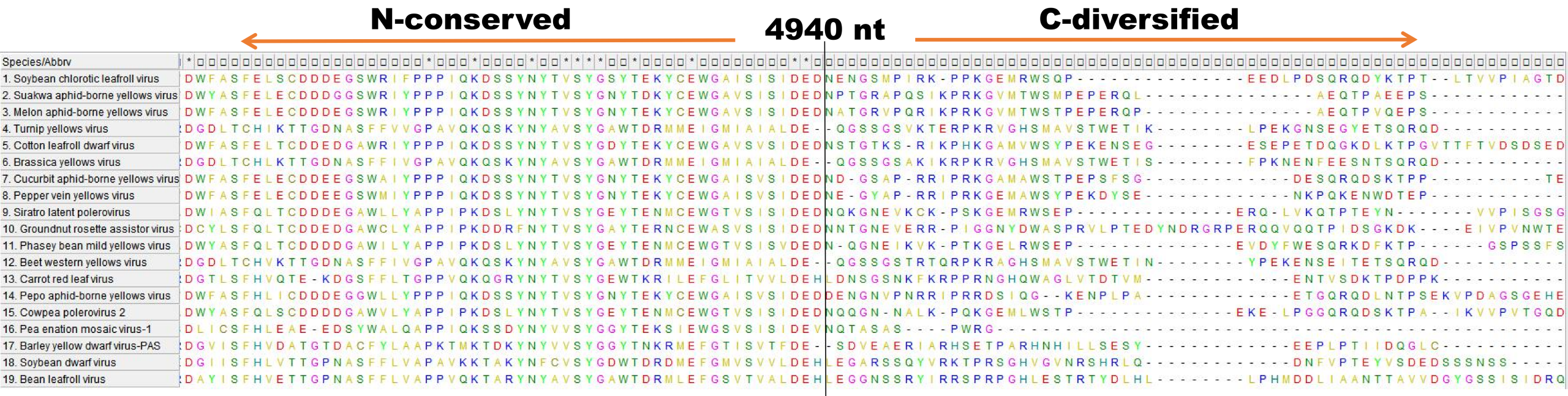

Supplement: Supplementary file 1 [file viruses-14-01428-s001.zip › viruses-1783399-figure S2.pdf]
